# Ovarian Metabolic activity in Dehydroepiandrosterone-Induced Polycystic Ovary in Wistar rats Treated with Aspirin

**DOI:** 10.5935/1518-0557.20190059

**Published:** 2020

**Authors:** Olugbemi T Olaniyan, Okoli Bamidele, Silas Uche, Adebayo Femi, Dare Ayobami, Oluwafemi Ayoola, Modupe Builders, Pratap Chand Mali

**Affiliations:** 1 Laboratory for Reproductive Biology and Developmental Programming, Department of Physiology, Edo University Iyamho, Edo State, Nigeria; 2 Institute of Chemical and Biotechnology, Vaal University of Technology, Southern Gauteng Science and Technology Park, Sebokeng, South Africa; 3 Department of Physiology, Bingham University Karu, Nasarawa State, Nigeria; 4 Pan African School of Health Technology, Offa, Kwara State, Nigeria; 5 Department of Pharmacology, Faculty of Pharmacy, Bingham University Karu, Nigeria; 6 Reproductive Biomedicine and Natural Product Lab, Department of Zoology, University of Rajasthan, Jaipur, India

**Keywords:** polycystic ovary syndrome, aspirin, dehydroepiandrosterone, reproduction, Wistar rat

## Abstract

**Objectives:**

Polycystic ovary syndrome (PCOS) represents 75% of the cases of anovulatory infertility. The aim of this study was to investigate the role of aspirin on dehydroepiandrosterone (DHEA) - induced polycystic ovary syndrome in Wistar rats.

**Methods:**

Twenty eight (28) pre-pubertal female Wistar rats of 21 days old weighing 16 - 21 g were divided into 4 groups (7 rats/group) and treated as follows; group I received distilled water and served as Control; Group II received 6 mg/100 g body weight DHEA in 0.2 ml of oil subcutaneously to induce PCOS. Group III received 7.5 mg/kg of aspirin orally; Group IV received 6 mg/100kg of body weight of DHEA in 0.2ml of oil subcutaneously and 7.5 mg/kg of aspirin orally. After 15 days of administration, the rats were slaughtered by cervical dislocation. Blood samples and ovaries were collected for reproductive hormonal analysis, biochemical and histopathological analysis. The expressions of mRNA androgen receptor (AR) gene in the ovary were determined by real time reverse transcriptase polymerase chain reaction (qPCR). All the data was analyzed using one way ANOVA with the Graph pad prism software version 6. A *p*<0.05 was considered significant.

**Results:**

The results obtained showed that dehydroepiandrosterone treatment caused significant decrease (*p*<0.05) in total protein, superoxide Dismutase (SOD), glutathione-s- transferase (GST), Ca^2+^ ATPase, and significant increase (*p*<0.05) in malondialdehyde, vascular endothelial growth factor, tumor necrosis factor and estrogen as compared to Controls. The group co-administered with DHEA and aspirin showed significant increases in SOD, GST, CAT, GSH, Progesterone, Ca^2+^ ATPase, Na^+^ ATPase, H^+^ ATPase and significant reduction (*p*<0.05) in malondialdehyde, VEGF, TNF-α and estrogen as compared with the DHEA group. The histopathological analysis showed reductions in cystic fibrosis, atretic ovaries, increased expression of Bcl-2 and E- Cadherin and reduced Bax expression in the group that received Aspirin and DHEA.

**Conclusion:**

This study clearly demonstrates that Aspirin has ameliorating effects against polycystic ovary syndrome via anti-inflammatory and hormonal modulatory pathways.

## INTRODUCTION

Infertility is a condition of the reproductive system, defined by the failure to achieve a clinical pregnancy after 12 months or more of regular unprotected sexual intercourse (Rowe *et al*., 1993). It may be caused by an underlying medical condition that may damage the fallopian tubes, interfere with ovulation, or cause hormonal complications. Infertility resulting from ovarian dysfunction may be due to the absence of eggs in the ovaries or a complete blockage of the ovaries. Ovarian dystrophy (physical damage to the ovaries, or ovaries with multiple cysts) and luteinized unruptured follicle syndrome (LUFS), in which case the egg may have matured properly but the follicle failed to burst, or it burst without releasing the egg may occur and cause an anovulatory cycle (Azziz *et al.,* 2004). Polycystic ovary syndrome (PCOS) is usually a hereditary problem and accounts for up to 90% of anovulation cases (Barbieri, 2001). In PCOS the ovaries produce high amounts of androgens, particularly testosterone, and thus amenorrhea or oligomenorrhea is quite common.

The increased androgen production in PCOS results in high levels of luteinizing hormone (LH) and low levels of follicle-stimulating hormone (FSH), so that follicles are prevented from producing a mature egg. The main source of androgen in women with PCOS is the ovaries (Barnes *et al.,* 1989), although 36 - 50% of women with PCOS have elevated adrenal androgen such as dehydroepiandrosterone (DHEA) (Teede *et al*., 2010). Studies have shown that hyperandrogenization of mice with DHEA prevents ovulation by increasing ovarian oxidative stress, inflammation and altering the endocrine and immune systems (Belgorosky *et al.,* 2010).

Pharmacological interventions and antioxidants are considered treatment regimens for PCOS. Aspirin (acetylsalicylic acid, ASA) has been used as one of the most famous, inexpensive, easily available and widely used Non-steroidal Anti-inflammatory Drug (NSAID). Aspirin is used in different roles, such as anti-inflammatory, anti-platelets, analgesic and antipyretic (Al-Janabi *et al*., 2007). *In-vitro* and *in-vivo* studies show that aspirin in high doses causes death of blood vessel tissues (Smith *et al*., 2001). Aspirin inhibits continuous production of prostaglandin, which causes unopposed constriction of arterioles, resulting in ischemia of tubules and epithelial cell death (Yasmeen *et al*., 2008). The inhibitory activity of aspirin is also found on the endocrine hormones viz, adrenocorticotrophic hormone, endorphin, cortisol, prolactin and growth hormone via a possible stimulatory role of prostaglandin (Di Luigi *et al*., 2001).

Aspirin exerts its effect primarily by interfering with the biosynthesis of cyclic prostanoids, i.e. thromboxane A_2_ (TXA_2_), prostacyclin, and other prostaglandins. These prostanoids are generated by the enzymatically catalyzed oxidation of arachidonic acid, which is derived from membrane phospholipids. Arachidonic acid is metabolized by the prostaglandin (PG) H-synthase enzyme, which through its cyclooxygenase (COX) and peroxidase activities, results in the production of PGG2 and PGH2, respectively. PGH2 is then modified by specific synthases, thus producing prostaglandins D2E2, F2a, I2 (prostacyclin), and TXA2, all of which mediate specific cellular functions. PGH-synthase, also referred to as COX, exists in 2 isoforms that have significant homology of their amino acid sequences (Williams & DuBois, 1996). A single amino acid substitution in the catalytic site of the enzyme confers selectivity to the COX isoforms inhibitors (Gierse *et al*., 1996). The first isoform (COX-1) is expressed in the endoplasmic reticulum of most cells (Morita *et al*., 1995) and results in the synthesis of homeostatic prostaglandins responsible for normal cellular functions. The second isoform (COX-2) is not routinely present in most mammalian cells but, rather, it is rapidly inducible by inflammatory stimuli and growth factors, and results in the production of prostaglandins that contribute to the inflammatory response (Xie *et al*., 1991).

Chronic low-grade inflammation is considered an important feature of polycystic ovary syndrome and has been suggested to participate in the pathogenesis and development of PCOS (Alanbay *et al*., 2012). Therefore, this study's aim is to investigate the role of aspirin as a non-steroidal anti-inflammatory drug on dehydroepiandrosterone-Induced polycystic ovary syndrome in Wistar rats.

## MATERIALS AND METHODS

Twenty eight (28) pre-pubertal female Wistar rats of 21 days of age, weighing 16 - 21 g were obtained from the Department of Human Physiology, Faculty of Basic Medical Sciences, College of Medicine, University of Ibadan and were housed in cages in a well-ventilated animal house of Bingham University, Karu, Nasarawa State, Nigeria. They were provided with rat pellets and water *ad libitum.* The ethical approval on animal rights act was obtained from the Institutional Animal Care Committee of the same University. All the experimental procedures were done following the experimental guidelines of the Institutional Animal Ethics Committee (IAEC) of Bingham University, Karu, Nasarawa State, Nigeria.

### Animal treatment and tissue collection

Twenty-eight immature female Wistar rats (21 days old) were randomly divided into four groups (n=7): The rats in group I served as Control and were given distilled water daily, group II animals were injected with DHEA (6 mg/100 g in 0.2 ml corn oil subcutaneously daily) to induce PCOS, group III was administered with Aspirin (7.5 mg/kg orally daily) and group IV was injected with DHEA (6 mg/100 g in 0.2 ml corn oil subcutaneously) and 7.5 mg/kg of Aspirin orally respectively. All treatments lasted 15 days. Twenty-four hours after the last day of administration, blood samples were collected via the retro-orbital venous sinus (Van Herck *et al.,* 1992) and serum was obtained for the determination of female sex hormones (Progesterone and estrogen). The rats were then slaughtered by cervical dislocation. The ovaries were harvested and cleaned of adherent connective fat tissue for further biochemical studies. The ovaries were rapidly kept in RNA and stored at 4ᵒC until used for the determination of mRNA and androgen receptor gene expression, and one ovary was fixed in bouins fluid and processed for histopathological analysis.

### Study Procedures

#### Antioxidant/oxidative stress

##### Lipid peroxidation assay

Malondialdehyde (MDA) levels were estimated by the method described by Kartha & Krishnamurthy (1978). Malondialdehyde was measured as an indicator of lipid peroxidation and ROS by extension. Samples were placed in a micro-centrifuge tube and incubated with thiobarbituric acid (TBA). Following incubation, the samples were centrifuged (2000 rpm, 10 minutes) and the absorbance of the pink clear supernatant was measured at 532 nm in duplicate samples. Malondialdehyde bis-(dimethyl acetal) was used as the external standard. Thiobarbituric acid reactive substances were expressed in terms of nano moles of MDA/gram of wet tissue. Lipid peroxidation was determined by measuring the thiobarbituric acid reactive substances (TBARS) produced during lipid peroxidation (Varshney & Kale, 1990).

##### Determination of tissue superoxide dismutase activity

Superoxide dismutase (SOD) was estimated by the technique explained by Beauchamp & Fridovich (1971). The activity was expressed as unit/mg of protein. The level of SOD activity was determined by the method described by Misra & Fridovich (1972). SOD's ability of to inhibit the autoxidation of epinephrine at pH 10.2 makes this reaction the basis for a simple assay for superoxide dismutase. The superoxide (O2 •−) radical generated by the xanthine oxidase reaction causes the oxidation of epinephrine to adrenochrome and the yield of adrenochrome produced per introduced O2 •− increases with pH (Valerino & McCormack, 1971) and with the concentrations of epinephrine. These results led to the idea that epinephrine autoxidation happens by at least two distinct pathways, only one of which is a free radical chain reaction involving the superoxide (O2 •−) radical, and hence subject to inhibition by SOD.

##### Determination of tissue catalase activity

Catalase activity was determined according to the method described by Sinha (1972). This method is based on the fact that dichromate in acetic acid is reduced to chromic acetate when heated in the presence of H_2_O_2_ with the formation of perchromic acid as an unstable intermediate. The chromic acetate then produced is measured by colorimetric analysis at 570 - 610 nm. Since dichromate has no absorbency in this region, the presence of the compound in the assay mixture does not interfere with the colorimetric determination of chromic acetate. The catalase preparation is let to split H_2_O_2_ for different periods of time. The reaction is stopped at a particular time with the addition of a dichromate/acetic acid mixture and the remaining H_2_O_2_ is determined by measuring chromic acetate by colorimetric analysis after heating the reaction mixture.

##### Determination of tissue glutathione-S-transferase activity

Glutathione-S-transferase (GST) activity was determined following the method described by Habig *et al.* (1974). The method is based on the principle that glutathione-S-transferase demonstrates a relatively high level of activity in the presence of 1-chloro-2,4-dinitrobenzene (CDNB), the substrate used in the assay to measure GST activity. When CDNB is conjugated with reduced glutathione, the maximum absorption shifts to a longer wavelength. The absorption increase at the new wavelength of 340 nm provides a direct measurement of the enzymatic reaction.

##### Reduced glutathione assay (GSH)

Reduced glutathione was estimated by the method of Jollow *et al.* (1974) by using 1, 2-dithio-bis nitro benzoic acid (DTNB) as substrate. The yellow color developed was read immediately at 412 nm and expressed as µmol GSH/g tissue.

##### Total protein (Biuret reagent)

Protein content of the tissue samples was determined using the method described by Lowry *et al.* (1951).

##### Assay procedure

In alkaline medium, copper reacts with the peptide bonds of proteins to form a characteristic pink to purple biuret complex. Potassium sodium tartrate prevents the precipitation of copper hydroxide, and potassium iodide prevents the auto reduction of copper.

Protein + Cu^2Alkaline pH^> = Cu - Protein complex

The color intensity is directly proportional to the protein concentration. It is measured based on the increase in absorbance at 546 nm using spectrophotometry (F93 Drawell fluorospectrophotometer).

#### Cytokines

##### Tissue vascular endothelial growth factor (VEGF) test

The ELISA kit used was the Sandwich-ELISA. The micro ELISA plate provided in this kit was pre-coated with an antibody specific to vascular endothelial growth factor (VEGF). Standards or samples were added to the micro ELISA plate wells and combined with the specific antibody. Then a biotinylated detection antibody specific for VEGF and Avidin-Horseradish Peroxidase (HRP) conjugate were added successively to each micro plate well and incubated. Free components were washed away. Then substrate solution was added to each well. Only those wells that contain VEGF, biotinylated detection antibody and Avidin-HRP conjugate appeared blue in color. The enzyme-substrate reaction was terminated by the addition of stop solution and the color turned yellow. The optical density (OD) was measured spectrophotometrically at a wavelength of 450 nm. The OD value is proportional to the VEGF concentration. The VEGF concentration was calculated in the samples by comparing the OD of the samples to the standard curve.

##### Tissue Tumor Necrosis Factor Alpha (TNF-α) Test

The Enzyme-Linked Immunosorbent Assay (ELISA) kit was used in the study. TNF-α was added to the wells, pre-coated with the TNF-α monoclonal antibody. After incubation, a biotin-conjugated anti-Rat TNF-α antibody was added, and it binds to the Rat TNF-α. After incubation unbound biotin-conjugated anti-Rat TNF-α antibody was washed away during the washing step. Streptavidin-HRP was added, and it binds to the biotin-conjugated anti-Rat TNF-α antibody. After incubation, unbound Streptavidin-HRP was washed away during the washing step. Substrate solution was then added and color develops in proportion to the amount of Rat TNF-α. The reaction was terminated adding acidic stop solution, and the absorbance was measured at 450 nm.

##### Estimation of serum progesterone and estrogen levels

The serum samples obtained were analyzed to determine the concentrations of progesterone and estrogen. The analysis was carried out via the tube-based enzyme immunoassay (EIA) method. The protocol used in hormone testing followed the method described by the kit manufacturer (Immunometrics Limited UK) and met the WHO research program standards for reproductive studies.

##### Determination of ovarian proton pump (ATPase) activity

Na^+^/K^+^-ATPase, Calcium ATPase, and Hydrogen ATPase activities were analyzed based on a modification of the method published by Evans (1969). Spectrophotometer was used to measure the levels of inorganic phosphate in the ovarian tissue homogenate as per the method described by Bonting (1970).

#### Standard Phosphate curve

##### Reagents for Proton Pump Bioassay (ATPase)

1. 10 mM Na_2_HP0_4_

0.142 g of disodium hydrogen phosphate (BDA Chemicals Co, Ltd, England) was dissolved in a little quantity of distilled water and completed up to the mark in a 100 ml standard volumetric flask.

2. 1.25% Ammonium Molybdate in 6.5% H_2_S0_4_

6.25 g of Ammonium molybdate (NH_4_)_6_ M0_7_0_24_.4H_2_0 (Hopkins and Williams Ltd; England) was dissolved in 500ml of 6.5% sulphuric acid (BDH Chemicals ltd, England). The latter was prepared by mixing 32.5 ml of concentrated sulphuric acid in water and making up the solution to 500 ml in a standard volumetric flask. The reagent was then stored at room temperature in a plastic bottle.

3. 9% Ascorbic acid

22.5 g of L-ascorbic acid (Sigma Chemical Co; USA) was dissolved in distilled water in a 250 ml standard flask. The solution was then stored in a brown reagent bottle and kept in the refrigerator at 4ºC.

#### Proton Pump Bioassay (ATPase) Procedure

1 mM Na_2_HP0_4_ was used as the standard curve for determining the inorganic phosphate released. The procedure was adopted from Stewart (1974), which is based on a color reaction developed using 1.25% NH_4_ molybdate and 9% Ascorbic acid. The protocol for inorganic phosphate determination was followed according to the procedure described by Fiske & SubbaRow (1925).

#### Na ^+^/K^+^ ATPase activity determination in the ovarian homogenate

##### Enzyme activity (µmole pi/mg protein/hour x 10^-3^) expression

###### Reaction mix

0.5 mls of each of 0.35M of sodium chloride, 17.5 potassium chloride, 21.0 mM Magnesium chloride, 10 mM of Tris HCl at PH 7.4 and 8.0 mM Disodium ATP were mixed together in a test tube. We then added 0.2 mls of tissue homogenate to it and incubated at 37ºC for 60 minutes. The reaction was terminated by the addition of 0.8 ml of ice cold 10% (w/v) trichloroacetic acid (TCA), it was allowed to stand for 20 minutes at 4ºC. It was then centrifuged at 4000 rpm for 5 minutes. 1 ml of supernatant was then added to 1 ml of 25% ascorbic acid, it was kept at room temperature for 20 minutes and the absorbance was measured at 725 nm using the spectrophotometer, according to the method of Bonting (1970) and the enzyme was assayed by the Evans modified method (1969).

#### Ca^2+^ ATPase activity determination in the ovarian homogenate

##### Enzyme activity (µmole pi/mg protein/hour x 10^-3^) expression

###### Reaction mix

0.5 ml of each of 21.0 mM Magnesium chloride, 17.5 mM Calcium Chloride, 10 mM of Tris HCl at PH 7.4 and 8.0 mM Disodium ATP were mixed together in a test tube. 0.2 mls of tissue homogenate was added to it and incubated at 37ºC for 60 minutes. The reaction was terminated by the addition of 0.8 ml of ice cold 10% (w/v) trichloroacetic acid (TCA). It was allowed to stand for 20 minutes at 4ºC and then centrifuged at 4000 rpm for 5 minutes. 1 ml of supernatant was then added to 1 ml of 25% ascorbic acid, it was kept at room temperature for 20 minutes and the absorbance was measured at 725 nm, using the spectrophotometer according to the method described by Hjertén & Pan (1983) and the enzyme was assayed by the modified method of Evans (1969).

#### H^+^ ATPase activity determination in the ovarian homogenate

##### Enzyme activity (µmole pi/mg protein/hour x 10**^-3^**) expression

###### Reaction mix

0.5 mls of each of 21.0 mM Magnesium chloride, 17.5 mM potassium chloride, 10 mM of Tris HCl at PH 7.4 and 8.0 mM Disodium ATP are mixed together in a test tube. 0.2 mls of tissue homogenate were added to it and incubated at 37ºC for 60 minutes. The reaction was terminated by the addition of 0.8 ml of ice cold 10% (w/v) trichloroacetic acid (TCA), it was allowed to stand for 20 minutes at 4ºC, and then Centrifuge at 4000 rpm for 5 minutes. Take 1 ml of the supernatant, add 1 ml of 1.25% Ammonium molybdate and wait for 10 min. Then 1 ml of 9% ascorbic acid was added, kept at room temperature for 20 minutes and the absorbance was measured at 725 nm, using the spectrophotometer according to the method described by Ohnishi *et al*. (1982) and the enzyme was assayed by the modified method of Evans (1969).

#### mRNA Androgen receptor gene expression (qPCR)

##### RNA Extraction

The Total RNA Mini Kit was designed specifically for purifying total RNA from a variety of animal tissues. The samples were efficiently homogenized in a microcentrifuge tube using the provided micro pestle. Detergents and chaotropic salt were used to break the cells and inactivate RNase with an optional in-column DNase treatment. RNA in the chaotropic salt is bound by the glass fiber matrix of the spin column. As soon as the contaminants have been removed, using the Wash Buffer (containing ethanol), the purified total RNA was eluted by RNase-free Water.

##### Agarose Gel Electrophoresis

The extracted RNA was checked on 1% agarose gel electrophoresis. The gels were stained with Ethidium Bromide and visualized under Bluelight transilluminator.

##### Agarose gel electrophoresis preparation:

For a 10 cm x 10 cm minigel cast, 1% agarose gel was prepared by dissolving 0.4 g of agarose in 40 ml of 1x TAE buffer. The mixture of agarose and buffer was swirled gently to ensure complete dissolution. The colloidal solution formed was heated at medium heat in the microwave oven for 1-3 minutes or until a clear solution was obtained. The gel was allowed to cool to about 50ºC (the gel must not solidify) under running tap water. Precaution was taken to prevent this tap water from splashing into the gel. Ethidium Bromide was added at a concentration of 0.5 µg/ml (2 µl stock in 40 ml) and mixed by swirling till no trace of the stain is detected. The gel was poured into the gel tray set with combs and allowed to solidify. This usually takes about twenty minutes. After the gel has set, the casts are removed and the gel tray (with the solidified gel) is set in the gel tank, submerged in the running buffer (1x TAE). Afterwards, the gel comb is gently removed from the submerged gel and the amplicons were loaded in the wells created by the comb. The genomic RNA extracted from each of the samples is mixed with a 6x loading dye in the ratio of 5 µl of the sample/amplicon to 1 µl of loading buffer. This was combined individually and loaded into the wells.

##### cDNA Synthesis

1 µg of each of the extracted RNA samples was converted to cDNA using the Bioline SensiFAST cDNA synthesis kit (according to manufacturer's protocol). The reaction contains 1 µl of Reverse Transcriptase and 4 µl of 5x TransAmp Buffer. Nuclease Free Water was added to make the reaction volume up to 20 µl. The thermocycling conditions were as follows: annealing at 25ºC for 10 minutes, Reverse Transcription at 42ºC for 15 minutes, an additional 48ºC for 15 minutes because of the highly structured RNA and lastly, inactivation of the enzyme at 85ºC for 5 minutes.

##### Real Time Polymerase Chain Reaction

The synthesized cDNA was amplified using the Bioer LineGene 9600 Real Time PCR machine. This was done in 10 µl reactions consisting of 2 µl of the template, 2 µl of the Solis Biodyne 5x qPCR mix, 0.25 µl each of the forward and reverse primers and 5.5 µl of nuclease free water. This process was carried out for all the samples and we made a No Template Control reaction, in which water was substituted for the template. The qPCR conditions were as follows: initial activation at 95ºC for 12 minutes, Denaturation at 95ºC for 15 seconds, Annealing at 65ºC for 20 seconds and Elongation at 72ºC for 20 seconds. Same cycling conditions were used for the house keeping gene βActin, but the annealing temperature was changed to 64ºC. The electrophoresis was run at 75 volts for 1 hour, after which we viewed and photographed it under a blue light transilluminator. A Solis biodyne 100 bp DNA ladder was run alongside the extracted RNA samples.

##### Histological Techniques

Histological examination was carried out on the tissues fixed in Bouin's fluid. Tissue blocks were sectioned for routine Hematoxylin and Eosin (H&E). The fixed organs were cut in about 0.5 cm cross-sections and transferred to 70% alcohol for dehydration. The tissues were rinsed in 90% and absolute alcohol and xylene for different durations before they were transferred into two changes of molten paraffin wax for 1 hour each in an oven at 65ᵒC for infiltration. They were subsequently embedded and sliced in serial sections using a rotary microtome at six microns (6 µ). The tissues were transferred onto albumenized slides and allowed to dry on a hot plate for 2 minutes. The slides were dewaxed with xylene and passed through absolute alcohol (2 changes); 70% alcohol, 50% alcohol and then water for 5 minutes. The slides were then stained with hematoxylin and eosin.

##### Immunohistochemistry (Bax, BCl**_2_** and E-Cadherin)

Samples for immunohistochemical studies were fixed in 10% formalin, and then after dehydration and embedding in paraffin slice into 7 µm sections. To identify Bax, BCl_2_ and E-Cadherin proteins, preparations from the groups were used. For each preparation a negative Control was performed (a slide without primary antibody). The protein expression level was evaluated with a standard three-step immunohistochemical procedure. Rabbit Bax, BCl_2_ and E-Cadherin antibodies were used as a primary antibody. Then biotinylated secondary antibody was added, and then horse-radish peroxidase conjugated with streptavidin. Since streptavidin has a great affinity to biotin, it binds to the place where the primary antibody coats the background. After adding a chromatogen (DAB) a reddish color appears.

##### Data analysis

The results were expressed as Mean ± standard error of mean (SEM), and subjected to statistical analysis using the ANOVA Graph-Pad prism software version 6, Tukey post-hoc analysis for data analysis. A *p*<0.05 was considered significant.

## RESULTS

### Effects of Aspirin administration on the ovarian malondialdehyde level in DHEA-Induced polycystic ovary in Wistar rats

**Figure 1:**
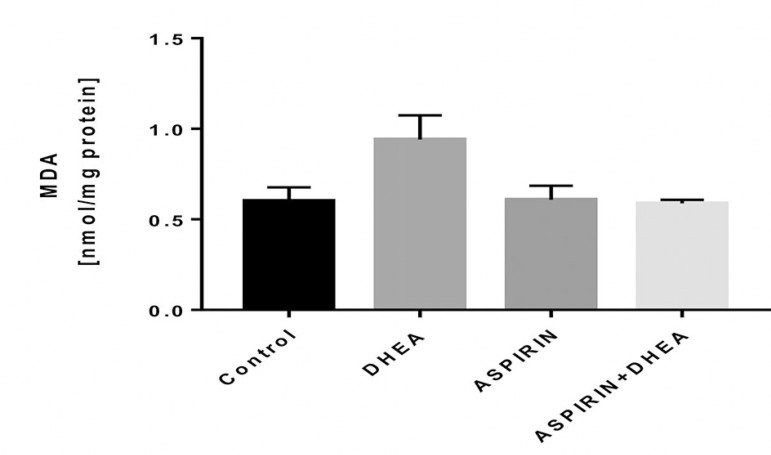
The effects of oral administration of 7.5 mg/kg Aspirin on malondialdehyde levels in Dehydroepiandrosterone-Induced polycystic ovaries in Wistar rats Values are presented as mean±SEM, n=7, + denote *p*<0.05 when compared with Control values *denote *p*<0.05 when compared with the DHEA-treated group, ** denote *p*<0.01 when compared with the DHEA-treated group

The results obtained showed that the group which received Aspirin and DHEA had a significant decrease (*p*<0.05) in MDA levels when compared with the DHEA group. The dehydroepiandrosterone-treated group showed a significant increase (*p*<0.05) in malondialdehyde levels when compared with the Control group (Figure 1).

### Effects of Aspirin administration on ovarian antioxidant levels in DHEA-Induced polycystic ovary in Wistar rats

The results showed that the DHEA group had a significant decrease (*p*<0.05) in antioxidant levels when compared with the Control Wistar rats. The group co-administered with Aspirin and DHEA showed an increase in the level of antioxidant as compared with the DHEA-treated group (Table 1).

**Table 1:** The effects of oral administration of 7.5 mg/kg Aspirin on antioxidant enzymes in Dehydroepiandrosterone-Induced polycystic ovaries in Wistar Rats

Groups	SOD U/ml/mg protein	CAT µmol/min/mg protein	GST µmol/min/mg protein	GSH mg/ml/mg protein
I	0.63±0.03	106.6±2.15	0.08±0.01	7.50±0.52
II	0.44±0.03^+^	93.0±0.39^+^	0.06±0.00^+^	6.09±0.48
III	1.57±0.08	102.0±6.09	0.07±0.00	6.48±0.54
IV	1.57±0.02*	105.3±3.41*	0.08±0.00*	8.52±0.27*

Values are presented as mean±SEM, n=7,

+ denote *p*<0.05 when compared with Control values

* denote *p*<0.05 when compared with DHEA-treated group,

SOD (Superoxide Dismutase),

CAT (Catalase),

GST (Glutathione-S-Transferase),

GSH (reduced glutathione).

### Aspirin administration effects on serum estrogen and progesterone levels in DHEA-Induced polycystic ovaries in Wistar rats

**Figure 2:**
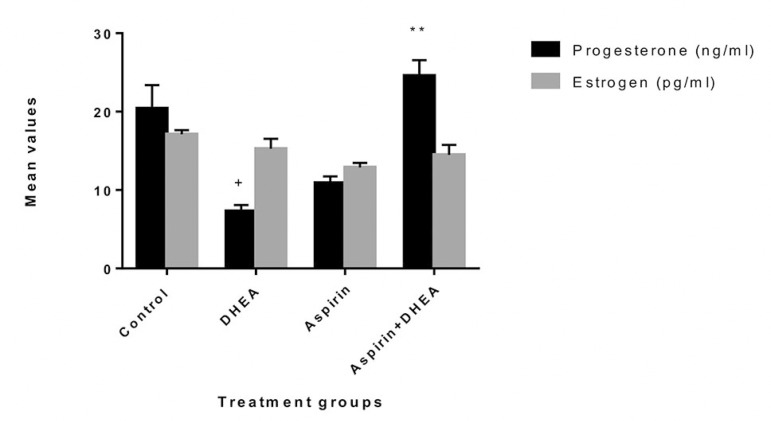
The effects of oral administration of 7.5 mg/kg Aspirin on progesterone and estrogen levels in Dehydroepiandrosterone-Induced polycystic ovaries in Wistar rats The values are presented as mean±SEM, n=7, + denote *p*<0.05 when compared with control values, *denote *p*<0.05 when compared with the DHEA-treated group, ** denote *p*<0.01 when compared with the DHEA-treated group

The results obtained showed that the DHEA-treated group had a significant decrease (*p*<0.05) in progesterone levels and a significant increase (*p*<0.05) in estrogen levels, as compared with the Control animals. The group co-administered with aspirin and DHEA had a significant increase (*p*<0.05) in progesterone levels when compared with the DHEA group (Figure 2).

### Effects of Aspirin administration on ovarian proton pump (ATPase) enzyme activity in DHEA-Induced polycystic ovaries in Wistar rats

The results obtained showed that the group co-administrated with aspirin and DHEA had a significant increase (*p*<0.05) in proton pump enzyme activity when compared with the DHEA group. In addition, the DHEA-treated group showed a reduction in the activity of these enzymes as compared with the Control group (Table 2).

**Table 2:** The effects of oral administration of 7.5 mg/kg Aspirin on ATPase enzyme activity in Dehydroepiandrosterone-Induced polycystic ovaries in Wistar Rats

Groups	Na^+^/K ^+^ATPase Piµmol/mg protein/hr/10^-3^	Ca^2+^ATPase Piµmol/mg protein/hr/10^-3^	H^+^ATPase Piµmol/mg protein/hr/10^-3^
I	0.49±0.04	4.72±0.19	0.44±0.00
II	0.41±0.01	4.02±0.06^+^	0.42±0.01^+^
III	0.46±0.01	4.38±0.14	0.44±0.01
IV	0.54±0.02*	4.34±0.11*	0.53±0.01*

Values are presented as mean±SEM, n=7,

+ denote *p*<0.05 when compared with Control values

* denote *p*<0.05 when compared with DHEA treated group.

### Effects of Aspirin administration on ovarian inflammatory cytokine levels in DHEA-Induced polycystic ovaries in Wistar rats

**Figure 3:**
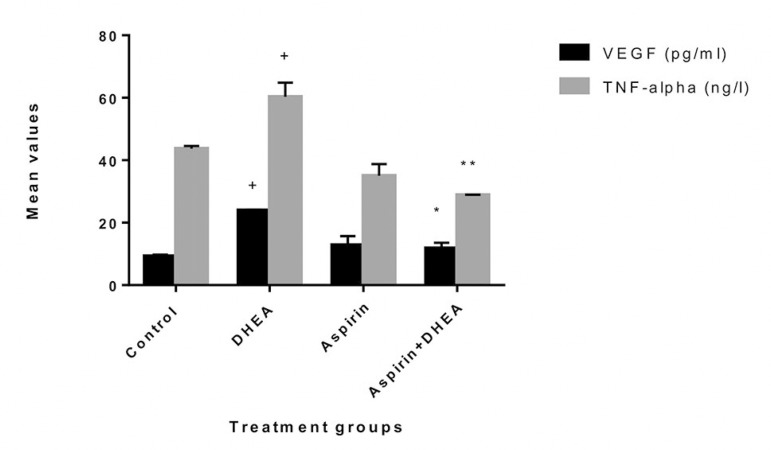
Effects of the oral administration of 7.5 mg/kg Aspirin on VEGF and TNF-α levels in Dehydroepiandrosterone-induced polycystic ovary in Wistar rats. Values are presented as mean±SEM, n=7, + denote *p*<0.05 when compared with Control values *denote *p*<0.05 when compared with the DHEA-treated group, **denote *p*<0.01 when compared with the DHEA-treated group

The results obtained showed that the DHEA-treated group caused a significant increase (*p*<0.05) in TNF-α and VEGF levels when compared with the Control group. However, the group co-administered with aspirin and DHEA had a significant decrease (*p*<0.05) in inflammatory cytokines levels when compared with the DHEA group (Figure 3).

### Effects of Aspirin administration on the granulosa cell mRNA Androgen receptor gene expression levels in DHEA-Induced polycystic ovaries in Wistar rats

**Figure 4:**
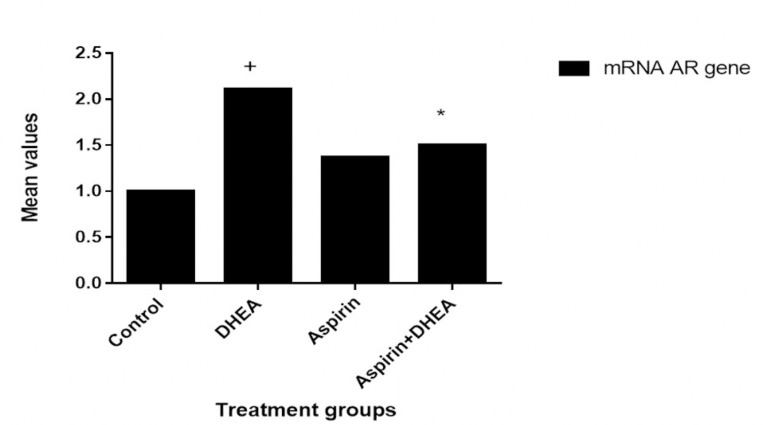
mRNA androgen receptor gene fold Change value (2^-ddCt) for aspirin treatment Values are presented as mean±SEM, n=7, + denote *p*<0.05 when compared with the Control value * denote *p*<0.05 when compared with DHEA-treated group

**Figure 5:**
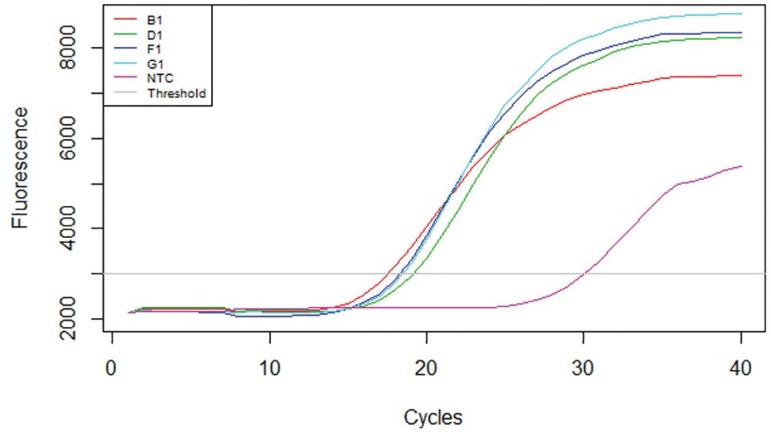
Amplification curve for the Androgen receptor treated with Aspirin

**Figure 6:**
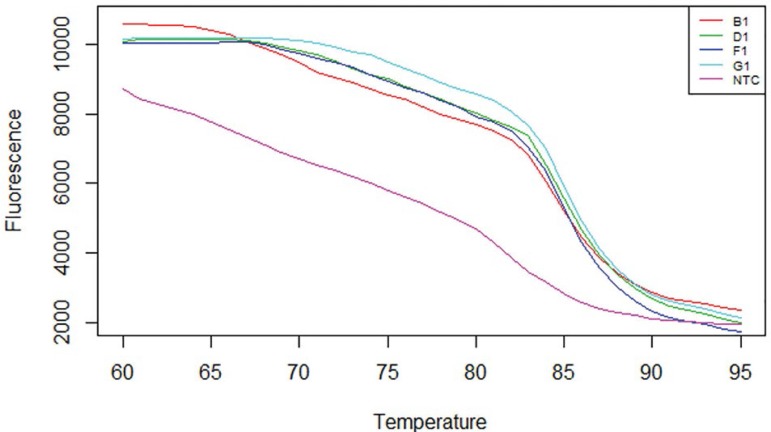
Melt Curve for androgen receptor treated with Aspirin.

The results obtained showed that the DHEA-treated group showed a significant expression (*p*<0.05) of the mRNA androgen receptor gene in the ovarian granulosa cells when compared to the Control group. However, the group co-administered with Aspirin and DHEA had a significant decrease (*p*<0.05) in the mRNA androgen receptor gene expression as compared to the DHEA group (Figures 4, 5 and 6, Table 3).

**Table 3:** List of primers and probe sequences

Ar	
RNAR F	ATGCTGGGCCTGTAGCCCCCT
RNAR R	CAGGCAGGTCTTCTGGGGTGGG
**B-Actin**	
RNACTB F	CCTCCGTCGCCGGTCCACACC
RNACTB R	TCTTGCTCTGGGCCTCGTCGC

## DISCUSSION

The polycystic ovary syndrome (PCOS) is an endocrine disorder of women at reproductive age, which is a major cause of anovulatory infertility (Joham *et al*., 2015). With regards to endometrial and ovarian cancer, several studies have reported a reduced risk of endometrial and ovarian cancer with the use of aspirin and other non-steroidal anti-inflammatory drugs (Drake & Becker, 2002; Fortuny *et al*., 2007). Modugno *et al*. (2001) described several possible pathways, through which NSAIDs might prevent endometrial and ovarian cancer, including via mechanisms reducing COX-2, inhibiting aromatase, re-oxygenating injured tissues and reducing TNF-α expression. Although treatment with aspirin or other anti-inflammatory agents is not the standard practice for treating polycystic ovary syndrome, emergent research recognizes the pro-inflammatory state often inherent in polycystic ovary syndrome and also the potentially beneficial effects of anti-inflammatory agents. In this study, the DHEA-treated group had a significant increase (*p*<0.05) in malondialdehyde (MDA) levels when compared to the Control group. On the other hand, there was a significant decrease (*p*<0.05) in MDA levels in the group co-administered with aspirin and DHEA (Figure 1).

Aspirin is considered to have anti-inflammatory properties due to its ability to suppress inflammatory cytokines known to induce oxidative damage in cells. In the present study, there were significant decreases (*p*<0.05) in superoxide dismutase (SOD), catalase, glutathione S transferase and reduced glutathione activity in the DHEA-treated group as compared with the Control group. This is in agreement with the study carried out by Zhang *et al*. (2004). This systematic decrease in antioxidant enzyme activity may be due to the augmented production of reactive oxygen species (ROS). It was reported that oxidative stress in polycystic ovary syndrome induces pro-inflammatory states that may contribute to co-morbidities, such as obesity, endothelial dysfunction and hyperandrogenism (Alanbay *et al*., 2012). Catalase is an intracellular antioxidant enzyme that is mainly located in cellular peroxisomes and to some extent in the cytosol, where it catalyzes the reduction of hydrogen peroxide to water and molecular oxygen. The group co-administered with aspirin and DHEA had significantly increased (*p*<0.05) antioxidant enzyme activity as compared with the DHEA group (Table 1). Glutathione, a tripeptide, is an important antioxidant present in millimolar concentrations in the ovary, and it plays the role of an intracellular scavenger.

This result is in agreement with the study done by Sabuncu *et al*. (2001), which reported that GSH was significantly lower in the PCOS group than in the Control group, and proposed that the lower levels of GSH may have been partly associated with insulin resistance. Aspirin helped trigger the increase in GSH on co-administration with DHEA, thereby increasing the intracellular scavenger activity. The glutathione-S-transferase is a group of multifunctional proteins, which play a central role in detoxification of electrophylic chemicals and the hepatic removal of potentially harmful hydrophobic compounds in the cell (Halliwell & Gutteridge, 2007). The rise in GST activity could be due to its induction to counter increased oxidative stress. In this study, we found that progesterone levels decreased significantly (*p*<0.05) in the DHEA-treated group when compared to Controls. Also there was a significant increase (*p*<0.05) in progesterone level in the group co-administered with aspirin and DHEA when compared to the DHEA group. The estrogen produced in PCOS is not balanced by the opposing effects of progesterone, which is produced after normal ovulation. Estrogen levels decreased in the DHEA-treated group when compared to Controls (Figure 2).

Several studies on the effects of low dose aspirin on estrogen have suggested the possible mechanism by which aspirin reduces tumor through its effect on estrogens, probably mediated through interference with estrogen synthesis via reduction in inflammation (Al-Janabi *et al.,* 2007; Duggan *et al.,* 2014; Spranger *et al.,* 1989). Aspirin may do so through direct tissue effects, or through pathways other than estrogens. They suggested that the use of aspirin at low doses for 6 months resulted in no change in serum estradiol, estrone, free estradiol, bioavailable estradiol, or SHBG in postmenopausal women. This is agreement with our results, as we found no changes in the levels of estrogen in PCOS rats treated with aspirin. It is well established that prolonged exposure to unopposed estrogen in the absence of sufficient progesterone, which is induced by anovulation, is also regarded as a major factor causing hyperplasia and cancer formation in PCOS (Dunaif *et al*., 1996). Estrogen could bind to its nuclear receptor, stimulating secretions of various growth factors such as Insulin-like Growth Factor (IGF), epidermal growth factor (EGF) and vascular endothelial growth factor (VEGF), thereby activating Extracellular signal-regulated kinases (ERK), a signaling pathway to promote endothelial and ovarian proliferation, and even cancer formation (Driggers & Segars, 2002). In addition, estrogen metabolites could also be the inducer of ovarian and endometrial cancer by binding to DNA, causing further DNA damage associated with oxidative stress. Under oxidative stress, estrogen intermediate metabolites, including 2-hydroxyl estrone (2- OHEI), 4-hydroxyl estrone (4- OHEI) and 16 α-hydroxyl estrone (16α OHEI) could not be methylated and eliminated from the body, and would be oxidized to semiquinone compounds and quinone compounds (Driggers & Segars, 2002).

The two abnormal types of estrogen metabolites with electron affinity bound to the DNA's nucleophylic group by a covalent bond, causing DNA mutation and further leading to ovarian and endometrial cancer processes. In this study, we found that there was a significant increase (*p*<0.05) in the level of vascular endothelial growth factor (VEGF) and tumor necrosis factor (TNF-α) as compared to Controls - Figure 3. However, the group co-administered with aspirin and DHEA had significant decreases (*p*<0.05) in VEGF and TNF-α inflammatory cytokine and suppression of androgen-receptor gene in the granulosa cells of the ovary as per found in this study when compared with the DHEA-group. It is clear that aspirin has the capacity to modulate immune responses and have potential anti-inflammatory capacity with suppression of the androgen-receptor gene expression responsible for the hyperandrogenism during folliculogenesis as per indicated in this study (Figures 4, 5 and 6; Table 3).

Chronic inflammation is considered an important feature of polycystic ovary and has been suggested to participate in the pathogenesis and development of PCOS (Alanbay *et al*., 2012). Inflammatory markers, such as tumor necrosis factor (TNF) and interleukin-6 (IL-6) increased in women with PCOS, when compared with normal subjects (Alanbay *et al*., 2012). It has been accepted that there is a tight link between oxidative stress and inflammation, and it is hard to distinguish inflammation from oxidative stress - they usually come together (Siti *et al*., 2015). Oxidative stress and inflammation seem to contribute to hyperandrogenemia in PCOS, by enhancing the activities of ovarian steroidogenesis enzymes, which could stimulate androgen generation (Yang *et al*., 2011). Tumor necrosis factor (TNF-α), an inflammatory marker associated with tissue inflammation, has the ability to promote the proliferation of mesenchymal cells of the follicular membrane and the synthesis of androgen in rats (Spaczynski *et al*., 1999). In this study, there was a significant increase (*p*<0.05) in the proton pump ATPase enzyme activity (Na^+^/ K^+^ ATPase, Ca^2+^ and H^+^ ATPase) in the group co-administered with aspirin and DHEA when compared to the DHEA group as shown in Table 2.

Several studies have implicated decreased proton pump (ATPases) activity in female reproductive dysfunction. Reactive oxygen species (ROS) have been suggested to be a major contributing factor to ATPase activity impairment (Olaniyan *et al.,* 2018). ATPase has been shown to be very susceptible to free radicals, membrane lipid peroxidation (Misra *et al.,* 1998) and inflammation. Lipid peroxidation has been shown to alter Na^+^/K^+^-ATPase, calcium ATPase and magnesium ATPase functions by modification at specific active sites in a selective manner (Qayyum *et al.,* 2001). Depletion of glutathione and other protective antioxidants by ROS may greatly contribute to increased levels of reactive species, which may also account for impaired Na^+^/K^+^-ATPase activity (D'Ambrosio *et al.,* 2001).

The histology and Immunohistochemistry of the ovary photomicrograph section in the DHEA exposed rats show an increasing number of cystic follicles with a thicker theca cell layer, a marked higher level of collagen especially in the region around the follicle, there is absence of corpora lutea and increased expression of the Bax apoptotic protein. The group treated with aspirin shows a normal ovary histological architecture, and the interstitial tissues are also normal. While the group treated with both aspirin and DHEA revealed a decreased number of cystic follicles with a thinner theca cell layer, a reduced level of collagen, especially in the region around the follicle, increased expression of the BCl_2_ anti-apoptotic protein and the E-Cadherin adhesion molecule (Plates A-P).

**PLATES A–D:**
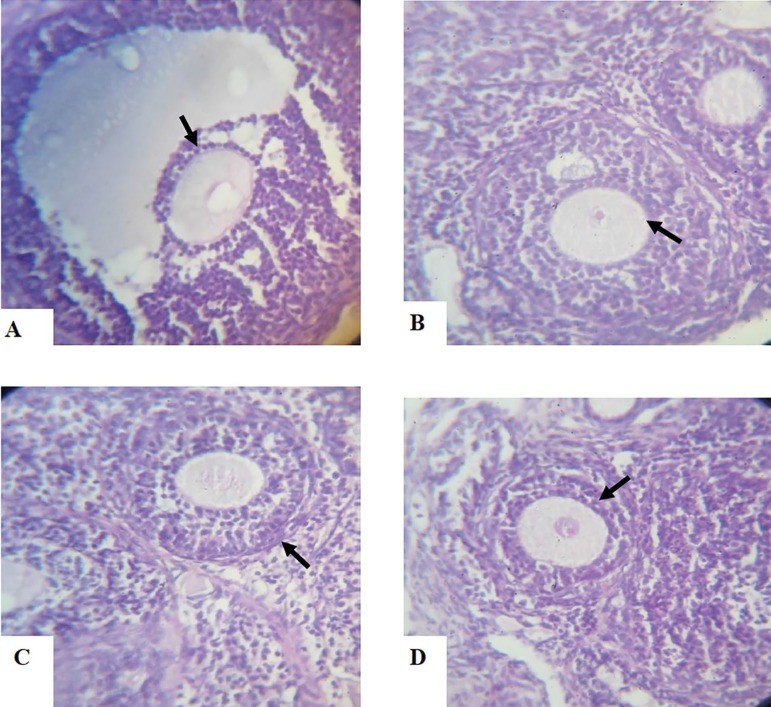
Histological sections of ovaries from Dehydroepiandrosterone (DHEA)-exposed rats and Controls following a 15-day treatment with aspirin using hematoxylin and eosin stain (H&E) X40 magnification **A.** Photomicrograph section of a Control-Group ovary showing normal follicle of different stages with well-organized surface epithelium, but without cystic follicle. The stromal cells of the ovarian follicles and corpus luteum are also well developed. **B.** Photomicrograph section of ovary from DHEA-exposed rats showing an increased number of cystic follicles with a thicker theca cell layer as indicated by the arrow, and a marked higher level of collagen, especially in the region around the follicle. There is absence of corpora lutea and antral follicles. **C.** Photomicrograph section of ovary from Aspirin-exposed rats showing normal histo-architecture of the ovary. The interstitial tissues are also normal. **D.** Photomicrograph section of ovary from Aspirin- and DHEA-exposed rats, showing a decreased number of cystic follicles with a thinner theca cell layer and a reduced level of collagen, especially in the region around the follicle.

**PLATES M-P:**
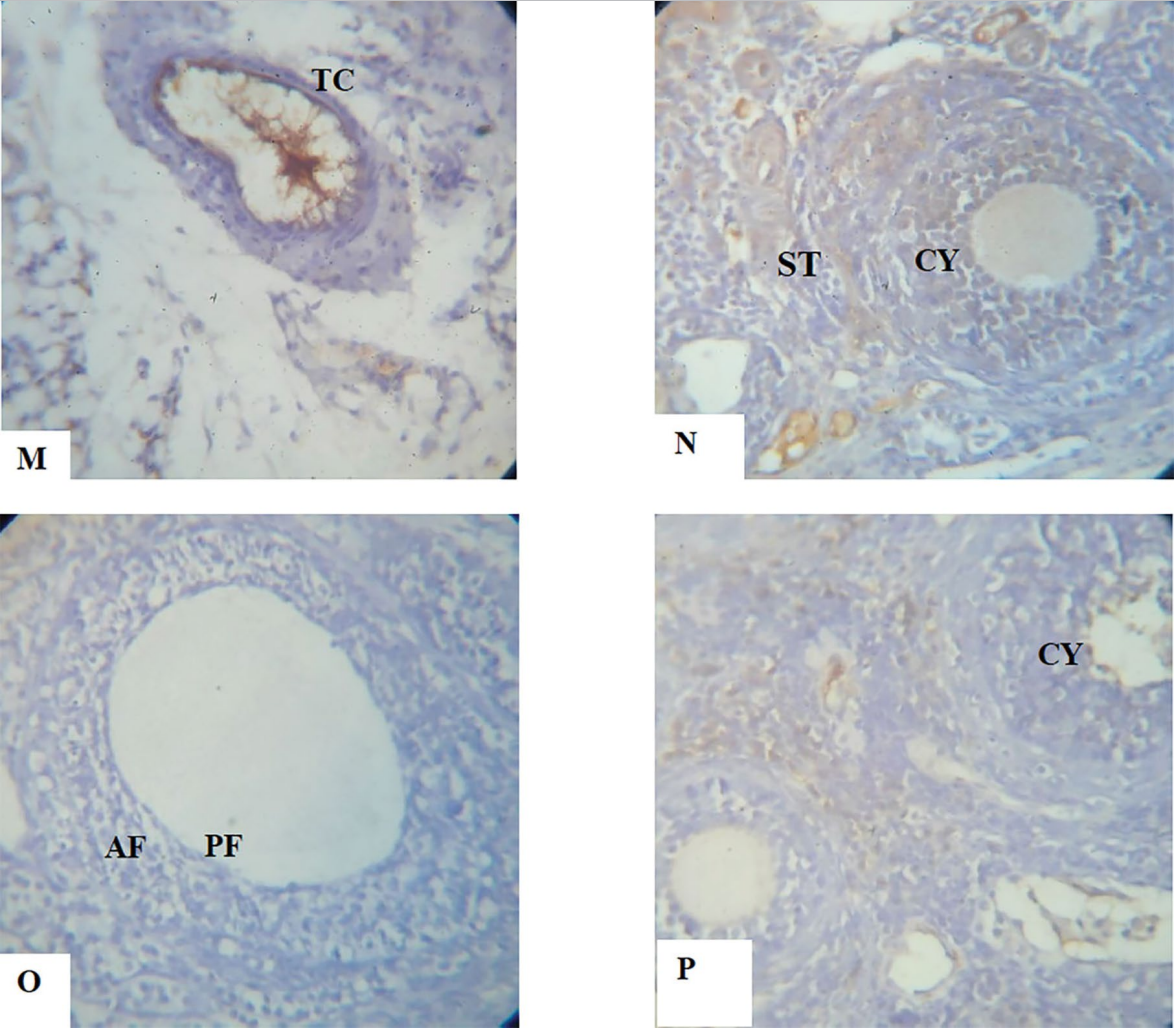
Show the immunohistochemical expression of the *apoptotic protein Bax, anti-apoptotic protein Bcl2* and Adhesion molecule E-Cadherin photomicrograph sections of ovaries from Dehydroepiandrosterone (DHEA) -exposed rats and Controls following a 15-day treatment with aspirin using X40 magnifications **M.** Photomicrograph section of ovary from the Control group showing normal expression of E-cadherin which was largely confined to areas of the interstitium, theca and surface epithelium. **N.** Photomicrograph section of ovary from DHEA-exposed rats, showing an increased number of cystic follicles (CY). Expression of E-Cadherin, which was largely confined to areas of the stroma, indicating a reduction in follicle growth. **O.** Photomicrograph section of an ovary from Aspirin-exposed rats showing normal preantral and antral follicles. The granulosa cells are also normal with well-expressed E-Cadherin, indicating follicle growth and development. **P.** Photomicrograph section of ovaries from Aspirin- and DHEA- exposed rats, showing a decreased number of cystic follicles. Expression of E-Cadherin was decreased in preantral and antral follicles, but well expressed in the granulosa cells. This indicates a significant improvement in ovary growth pattern.

## CONCLUSION

This study clearly demonstrates that Aspirin has ameliorating effects against polycystic ovary syndrome via anti-inflammatory and hormonal modulatory pathways.

## Figures and Tables

**PLATES E-H:**
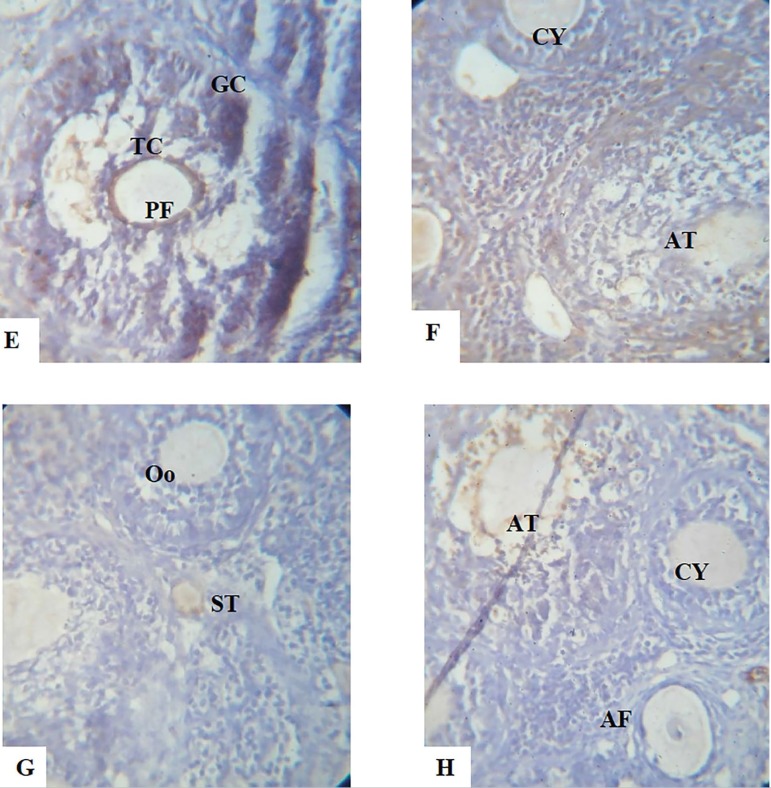
Show the immunohistochemical expression of the *apoptotic protein Bax, anti-apoptotic protein Bcl2* and Adhesion molecule E-Cadherin photomicrograph sections of ovaries from Dehydroepiandrosterone (DHEA) -exposed rats and Controls following a 15-day treatment with aspirin using X40 magnifications **E.** Ovary photomicrograph section of the Control group showing normal preantral follicles (PF), granulosa cells (GC) and theca cells (TC). The Bax expression is mainly located in the granulosa cells of antral follicles. **F.** Photomicrograph section of ovary from DHEA-exposed rats showing an increased number of cystic follicles (CY) and atretic follicles (AT), the Bax expression is higher in preantral and antral follicles, indicating a polycystic condition. **G.** Photomicrograph section of ovary from Aspirin-exposed rats showing normal preantral, antral follicle and stroma (ST). The granulosa cells and Oocytes (Oo) are also normal. **H.** Photomicrograph section of ovaries from Aspirin- and DHEA- exposed rats showing a decreased number of cystic follicles. The Bax expression is decreased in preantral and antral follicles (AF). This indicates improvements in the ovary’s cyto-architecture.

**PLATES I-L:**
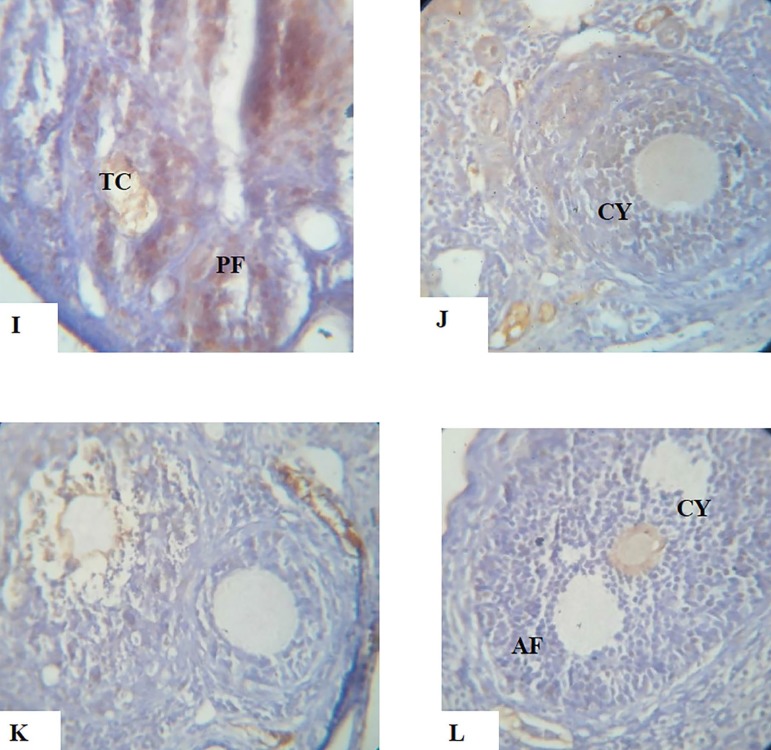
Show the immunohistochemical expression of the *apoptotic protein Bax, anti-apoptotic protein Bcl2* and Adhesion molecule E-Cadherin photomicrograph sections of ovaries from Dehydroepiandrosterone (DHEA) -exposed rats and Controls following a 15-day treatment with aspirin using X40 magnifications **I.** Photomicrograph section of ovary from Control group showing normal preantral follicles (PF), granulosa cells (GC) and theca cells (TC) with Bcl-2 expressions mainly localized in the granulosa cells of antral follicles. **J.** Photomicrograph section of ovary from DHEA-exposed rats showing an increased number of cystic follicles (CY). Bcl-2 protein was either absent in preantral follicles or weakly expressed in antral follicles, which are typical condition of polycystic ovary. **K.** Photomicrograph section of ovary from Aspirin-exposed rats showing normal preantral and antral follicle, with Bcl-2 expression mainly localized in granulosa cells of antral follicles. **L.** Photomicrograph section of ovary from Aspirin- and DHEA- exposed rats showing a decrease in number of cystic follicles. Bcl-2 expression was decreased in preantral and antral follicles (AF).

## References

[r1] Alanbay I, Ercan CM, Sakinci M, Coksuer H, Ozturk M, Tapan S (2012). A macrophage activation marker chitotriosidase in women with PCOS: does low-grade chronic inflammation in PCOS relate to PCOS itself or obesity?. Arch Gynecol Obstet.

[r2] Al-Janabi AS, Al-Zohyri AM, Al-Rubayai FK (2007). Pharmacological effects of low- dose of aspirin on ovulation rate in mature cycling female mice. Middle East Fertil Soc J.

[r3] Azziz R, Woods KS, Reyna R, Key TJ, Knochenhauer ES, Yildiz BO (2004). The prevalence and features of the polycystic ovary syndrome in an unselected population. J Clin Endocrinol Metab.

[r4] Barbieri RL (2001). The initial fertility consultation: recommendations concerning cigarette smoking, body mass index, and alcohol and caffeine consumption. Am J Obstet Gynecol.

[r5] Barnes RB, Rosenfield RL, Burstein S, Ehrmann DA (1989). Pituitary-ovarian responses to nafarelin testing in the polycystic ovary syndrome. N Engl J Med.

[r6] Beauchamp C, Fridovich I (1971). Superoxide dismutase: improved assays and an assay applicable to acrylamide gels. Anal Biochem.

[r7] Belgorosky D, Sander VA, Yorio MP, Faletti AG, Motta AB (2010). Hyperandrogenism alters intraovarian parameters during early folliculogenesis in mice. Reprod Biomed Online.

[r8] Bonting SL, Bittar EE (1970). Presence of Enzyme System in Mammalian Tissues. Membranes and Ion Transport.

[r9] D'Ambrosio SM, Gibson-D'Ambrosio RE, Brady T, Oberyszyn AS, Robertson FM (2001). Mechanisms of nitric oxide-induced cytotoxicity in normal human hepatocytes. Environ Mol Mutagen.

[r10] Di Luigi L, Guidetti L, Romanelli F, Baldari C, Conte D (2001). Acetylsalicylic acid inhibits the pituitary response to exercise-related stress in humans. Med Sci Sports Exerc.

[r11] Drake JG, Becker JL (2002). Aspirin-induced inhibition of ovarian tumor cell growth. Obstet Gynecol.

[r12] Driggers PH, Segars JH (2002). Estrogen action and cytoplasmic signaling pathways. Part II: The role of growth factors and phosphorylation in estrogen signaling. Trends Endocrinol Metab.

[r13] Duggan C, Wang CY, Xiao L, McTiernan A (2014). Aspirin and serum estrogens in postmenopausal women: a randomized Controlled clinical trial. Cancer Prev Res (Phila).

[r14] Dunaif A, Segal KR, Shelley DR, Green G, Dobrjansky A, Licholai T (1996). Evidence for distinctive and intrinsic defects in insulin action in polycystic ovary syndrome. Diabetes.

[r15] Evans Jr DJ (1969). Membrane adenosine triphosphatase of Escherichia coli: activation by calcium ion and inhibition by monovalent cations. J Bacteriol.

[r16] Fiske CH, SubbaRow Y (1925). The colorimetric determination of phosphorus. J Biol Chem.

[r17] Fortuny J, Johnson CC, Bohlke K, Chow WH, Hart G, Kucera G, Mujumdar U, Ownby D, Wells K, Yood MU, Engel LS (2007). Use of anti-inflammatory drugs and lower esophageal sphincter-relaxing drugs and risk of esophageal and gastric cancers. Clin Gastroenterol Hepatol.

[r18] Gierse JK, McDonald JJ, Hauser SD, Rangwala SH, Koboldt CM, Seibert K (1996). A single amino acid difference between cyclooxygenase-1 (COX-1) and -2 (COX-2) reverses the selectivity of COX-2 specific inhibitors. J Biol Chem.

[r19] Habig WH, Pabst MJ, Jakoby WB (1974). Glutathione S-transferases. The first enzymatic step in mercapturic acid formation. J Biol Chem.

[r20] Halliwell B, Gutteridge JMC (2007). Free Radicals in Biology and Medicine.

[r21] Hjertén S, Pan H (1983). Purification and characterization of two forms of a low-affinity Ca2+-ATPase from erythrocyte membranes. Biochim Biophys Acta.

[r22] Joham AE, Teede HJ, Ranasinha S, Zoungas S, Boyle J (2015). Prevalence of infertility and use of fertility treatment in women with polycystic ovary syndrome: data from a large community-based cohort study. J Womens Health (Larchmt).

[r23] Jollow DJ, Mitchell JR, Zampaglione N, Gillette JR (1974). Bromobenzene-induced liver necrosis. Protective role of glutathione and evidence for 3,4-bromobenzene oxide as the hepatotoxic metabolite. Pharmacology.

[r24] Kartha R, Krishnamurthy S (1978). Factors affecting invitro lipid peroxidation in rat brain homogenate. Indian J Physiol Pharmacol.

[r25] Lowry OH, Rosebrough NJ, Farr AL, Randall RJ (1951). Protein measurement with folin phenol reagent. J Biol Chem.

[r26] Misra HP, Fridovich L (1972). The role of superoxide anion in the auto oxidation of epinephrine and a simple assay for superoxide dismutase. J Biol Chem.

[r27] Misra RR, Smith GT, Waalkes MP (1998). Evaluation of the direct genotoxic potential of cadmium in four different rodent cell lines. Toxicology.

[r28] Modugno F, Ness RB, Wheeler JE (2001). Reproductive risk factors for epithelial ovarian cancer according to histologic type and invasiveness. Ann Epidemiol.

[r29] Morita I, Schindler M, Regier MK, Otto JC, Hori T, DeWitt DL, Smith WL (1995). Different intracellular locations for prostaglandin endoperoxide H synthase-1 and -2. J Biol Chem.

[r30] Ohnishi T, Suzuki T, Suzuki Y, Ozawa K (1982). A comparative study of plasma membrane Mg2+ -ATPase activities in normal, regenerating and malignant cells. Biochim Biophys Acta.

[r31] Olaniyan OT, Kunle-Alabi OT, Raji Y (2018). Protective effects of methanol extract of Plukenetia conophora seeds and 4H-Pyran-4-One 2,3-Dihydro-3,5-Dihydroxy-6-Methyl on the reproductive function of male Wistar rats treated with cadmium chloride. JBRA Assist Reprod.

[r32] Qayyum I, Zubrow AB, Ashraf QM, Kubin J, Delivoria-Papadopoulos M, Mishra OP (2001). Nitration as a mechanism of Na+, K+-ATPase modification during hypoxia in the cerebral cortex of the guinea pig fetus. Neurochem Res.

[r33] Rowe PJ, Comhaire FH, Hargreave TB, Mellows HJ, World Health Organization (1993). WHO Manual for the Standardized Investigation of the Infertile Couple.

[r34] Sabuncu T, Vural H, Harma M, Harma M (2001). Oxidative stress in polycystic ovary syndrome and its contribution to the risk of cardiovascular disease. Clin Biochem.

[r35] Sinha AK (1972). Colorimetric assay of catalase. Anal Biochem.

[r36] Siti HN, Kamisah Y, Kamsiah J (2015). The role of oxidative stress, antioxidants and vascular inflammation in cardiovascular disease (a review). Vascul Pharmacol.

[r37] Smith Jr SC, Blair SN, Bonow RO, Brass LM, Cerqueira MD, Dracup K, Fuster V, Gotto A, Grundy SM, Miller NH, Jacobs A, Jones D, Krauss RM, Mosca L, Ockene I, Pasternak RC, Pearson T, Pfeffer MA, Starke RD, Taubert KA (2001). AHA/ACC Scientific Statement: AHA/ACC guidelines for preventing heart attack and death in patients with atherosclerotic cardiovascular disease: 2001 update: A statement for healthcare professionals from the American Heart Association and the American College of Cardiology. Circulation.

[r38] Spaczynski RZ, Arici A, Duleba AJ (1999). Tumor necrosis factor-alpha stimulates proliferation of rat ovarian theca-interstitial cells. Biol Reprod.

[r39] Spranger M, Aspey BS, Harrison MJ (1989). Sex difference in antithrombotic effect of aspirin. Stroke.

[r40] Stewart DJ (1974). Sensitive automated methods for phosphate and (Na+ plus K+)-ATPase. Anal Biochem.

[r41] Teede H, Deeks A, Moran L (2010). Polycystic ovary syndrome: a complex condition with psychological, reproductive and metabolic manifestations that impacts on health across the lifespan. BMC Med.

[r42] Valerino DM, McCormack JJ (1971). Xanthine oxidase-mediated oxidation of epinephrine. Biochem Pharmacol.

[r43] van Herck H, Baumans V, Stafeu FR, Beynen AC (1992). A questionnaire-based inventory of the orbital puncture method in the Netherlands. Scand J Lab Anim Sci.

[r44] Varshney R, Kale RK (1990). Effects of calmodulin antagonist on radiation-induced lipid peroxidation in microsomes. Int J Radiat Biol.

[r45] Williams CS, DuBois RN (1996). Prostaglandin endoperoxide synthase: why two isoforms?. Am J Physiol.

[r46] Xie WL, Chipman JG, Robertson DL, Erikson RL, Simmons DL (1991). Expression of a mitogen-responsive gene encoding prostaglandin synthase is regulated by mRNA splicing. Proc Natl Acad Sci U S A.

[r47] Yang Y, Qiao J, Li R, Li MZ (2011). Is interleukin-18 associated with polycystic ovary syndrome?. Reprod Biol Endocrinol.

[r48] Yasmeen T, Yasmin F, Qureshi GS (2008). To evaluate the role of diclofenac sodium on renal parenchyma of young albino rats. Pak J Pharm Sci.

[r49] Zhang M, Lee AH, Binns CW (2004). Reproductive and dietary risk factors for epithelial ovarian cancer in China. Gynecol Oncol.

